# Combination therapy of tiotropium and ciclesonide attenuates airway inflammation and remodeling in a guinea pig model of chronic asthma

**DOI:** 10.1186/s12931-016-0327-6

**Published:** 2016-02-04

**Authors:** Loes E. M. Kistemaker, I. Sophie T. Bos, Mark H. Menzen, Harm Maarsingh, Herman Meurs, Reinoud Gosens

**Affiliations:** Department of Molecular Pharmacology, University of Groningen, A. Deusinglaan 1, 9713 AV Groningen, The Netherlands; GRIAC Research Institute, University Medical Center Groningen, University of Groningen, Groningen, The Netherlands; Department of Pharmaceutical Sciences, Gregory School of Pharmacy, Palm Beach Atlantic University, West Palm Beach, FL USA

**Keywords:** Anticholinergics, Inhaled corticosteroids, Interaction

## Abstract

**Background:**

The long-acting anticholinergic tiotropium has recently been registered for the treatment of asthma, and its use is associated with a reduction in exacerbation frequency. Anti-inflammatory and anti-remodeling effects of tiotropium have been demonstrated in in vitro and in vivo models. Because tiotropium treatment is used in combination with inhaled corticosteroids, potential additive effects between the two would be clinically relevant. Therefore, the aim of this study was to investigate additive effects between tiotropium and ciclesonide on airway inflammation and remodeling in guinea pig models of asthma.

**Methods:**

Guinea pigs (*n* = 3–8/group) were sensitized and challenged with ovalbumin in an acute (single challenge) and a chronic model (12 weekly challenges) of allergic asthma. Animals were treated with vehicle, nebulized tiotropium (0.01–0.3 mM) and/or intranasally instilled ciclesonide (0.001–1 mg/kg) before each challenge. Bronchoalveolar lavage fluid and lungs were collected for analysis of airway inflammation and remodeling.

**Results:**

Tiotropium and ciclesonide treatment, alone or in combination, did not inhibit airway inflammation in the acute asthma model. In a dose-finding study, low doses of tiotropium and ciclesonide inhibited airway eosinophilia and airway smooth muscle thickening in the chronic asthma model. Threshold doses of 0.01 mM tiotropium (nebulizer concentration) and 0.01 mg/kg ciclesonide were selected to investigate potential additive effects between both drugs. At these doses, tiotropium and ciclesonide did not inhibit airway eosinophilia or airway smooth muscle thickening when administered alone, but significantly inhibited these allergen-induced responses when administered in combination.

**Conclusions:**

Combined treatment with low doses of tiotropium and ciclesonide inhibits airway inflammation and remodeling in a guinea pig model of chronic asthma, suggesting that combined treatment with anticholinergics and corticosteroids may have anti-inflammatory and anti-remodeling activity in allergic airway diseases. Since tiotropium is registered as a therapy for asthma added on to corticosteroid treatment, these beneficial effects of the combination therapy may be clinically relevant.

**Electronic supplementary material:**

The online version of this article (doi:10.1186/s12931-016-0327-6) contains supplementary material, which is available to authorized users.

## Background

Asthma is a common obstructive airway disease, which currently affects around 300 million people worldwide and has a major debilitating impact on society [1]. In most cases, asthma is associated with an allergic response towards inhaled aeroallergens. Patients with asthma suffer from inflammation of the airways, causing hyperresponsiveness to specific and non-specific stimuli. This inflammation is characterized by an increase in eosinophils, CD4^+^ lymphocytes and T_H_2 cytokines including IL-4, IL-5 and IL-13 [2]. Moreover, remodeling of the bronchial tree is a significant pathology in severe asthma that contributes to airflow obstruction and loss of deep breath-induced bronchodilation [3]. Remodeling of the airways is characterized by increased extracellular matrix deposition in the subepithelial airway compartment and marked thickening of the bronchial smooth muscle. All these changes are associated with airflow limitation in severe asthma [4].

Current treatment for patients with asthma includes inhaled corticosteroids (ICS) and long-acting β_2_-agonists (LABA). Moreover, the long-acting muscarinic antagonist (LAMA) tiotropium has recently been registered for the treatment of asthma. Clinical trials have shown beneficial effects on lung function by addition of tiotropium to standard treatment in moderate and severe asthma patients [5–7]. In addition, treatment with tiotropium reduced the number of severe exacerbations [5], suggesting that tiotropium might exert anti-inflammatory effects in these patients.

Anti-inflammatory effects of anticholinergics have indeed been observed in in vitro and in vivo studies using various experimental models [8, 9]. In vitro, anticholinergics exert direct anti-inflammatory effects on inflammatory cells, including T cells [10] and macrophages [11], on epithelial cells [12], and on airway smooth muscle cells [13]. In addition, anticholinergics affect airway remodelling in vitro [9]. Muscarinic receptors regulate proliferation of airway smooth muscle cells [14] and fibroblasts [15], fibroblast to myofibroblast transition [16], and extracellular matrix deposition [17, 18]. These findings have been confirmed in in vivo animal models, demonstrating inhibitory effects of tiotropium or muscarinic M_3_ receptor knock-out on ovalbumin-induced inflammation and remodeling, including airway smooth muscle thickening, extracellular matrix deposition and mucus gland hypertrophy [19–22]. Effects of tiotropium on ovalbumin-induced inflammation and remodeling were comparable to the effects of the corticosteroid budesonide [22]. The effects of the combination of tiotropium and a corticosteroid on airway inflammation and remodeling are currently unknown. In vitro, it has been shown that the anticholinergic glycopyrrolate acts synergistically with budesonide in inhibiting TNF-α release from isolated monocytes [23], suggesting that the combination of anticholinergics and corticosteroids might be more effective than the monotherapies in vivo.

In view of the above mentioned beneficial effects of tiotropium and corticosteroids on allergic airway inflammation and remodeling, combination therapy with anticholinergics and corticosteroids might have additive protective effects on airway inflammation and remodeling. Therefore, in the present study, the effects of pre-treatment with tiotropium and ciclesonide on airway inflammation and remodeling were investigated using guinea pig models of acute and chronic asthma. Guinea pig models are valuable for the evaluation of pathophysiological mechanisms and pharmacological interventions in asthma, since the mechanisms underlying the allergic asthmatic reaction in guinea pigs are more comparable to humans, and therefore more physiologically relevant compared to commonly used rodent models [24]. In these guinea pig models, we demonstrate that tiotropium and ciclesonide do not inhibit *acute* allergen-induced inflammation, but do inhibit *chronic* allergen-induced airway inflammation and remodeling when applied in combination.

## Methods

### Animals

Outbred male, specified pathogen-free Dunkin Hartley guinea pigs (Harlan, Heathfield, UK), weighing 500–800 g, were used in this study. The animals were group-housed in individual cages in climate-controlled animal quarters and given water and food ad libitum, while a 12-h on/12-h off light cycle was maintained. All protocols described were approved by the University of Groningen Committee for Animal Experimentation (DEC6081).

### Ovalbumin administration

**Fig. 1:**
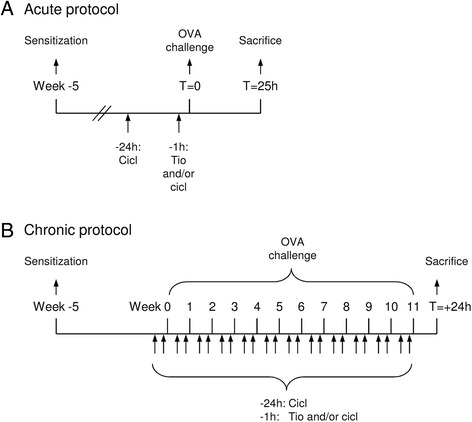
Experimental procedure. **a** acute protocol, **b** chronic protocol. Male Dunkin Hartley guinea pigs (*n* = 3–8 animals per group, see Additional file 1: Table S1-S3 for overview of groups) were sensitized to ovalbumin (OVA) by intraperitoneal injection of 0.5 ml allergen solution containing 100 μg/ml ovalbumin and 100 mg/ml Al(OH)_3_, and intracutaneous injection of 0.5 ml allergen solution. Subsequently, guinea pigs were challenged with OVA (0.05–0.1 %) via inhalation of aerosolized solution. Tiotropium (tio; 0.01–0.3 mM; 3 min inhalation time) was administered via aerosol inhalation and ciclesonide (cicl; 0.001–1 mg/kg) via intranasal instillation, 24 h and/or 1 h before every challenge. In the acute protocol, a bronchoalveolar lavage was performed 25 h after the ovalbumin challenge. In the chronic protocol, lungs were harvested for tissue sections 24 h after the last of 12 weekly OVA challenges

The animals were actively IgE-sensitized to ovalbumin as described previously [22]. In short, 0.5 ml of an allergen solution containing 100 μg/ml ovalbumin and 100 mg/ml Al(OH)_3_ in saline was injected intraperitoneally, while another 0.5 ml was divided over seven intracutaneous injection sites in the proximity of lymph nodes in the paws, lumbar regions, and the neck. The animals were used experimentally 5 weeks after sensitization (Fig. 1). Challenges with ovalbumin (0.05–0.1 % in saline; Sigma Chemical, St. Louis, MO) were performed by inhalation of aerosolized solutions until airway obstruction, as described previously [22]. The average ovalbumin dose to induce airway obstruction at the end of the protocol in the control group was 909 ± 1221 μg. The dose needed in the tiotropium and ciclesonide group was not different (302 ± 140 and 409 ± 259 μg respectively), whereas the dose was slightly higher in the group treated with the combination of tiotropium and ciclesonide (1759 ± 1326 μg). These differences in ovalbumin dose between the different groups were not statistically significant. Aerosols were produced by a DeVilbiss nebulizer (type 646; DeVilbiss, Somerset, PA) driven by an airflow of 8 l/min and resulting in an output of 0.33 ml/min. Provocations were carried out in a perspex cage (internal volume of 9 l) in which the guinea pigs could move freely.

### Drug administration

Tiotropium treatment (nebulizer concentration 0.01–0.3 mM in saline; Boehringer Ingelheim Pharma GmbH) was administered via inhalation of aerosolized solutions for 3 min, as described above for ovalbumin challenges. Treatment was performed 1 h prior to each ovalbumin challenge. Ciclesonide treatment was not possible via nebulization because of the poor solubility, and was therefore administered via intranasal instillation (0.001–1 mg/kg in saline containing 0.2 % Tween 80; Bufa BV). Conscious guinea pigs were held in an upright position, while 200 μl ciclesonide was slowly instilled intranasally. After the instilled solution was aspirated, the animals were kept in the upright position for an additional 2 min to allow sufficient spreading of the fluid throughout the airways. Ciclesonide treatment was performed 24 h and 1 h prior to each ovalbumin challenge. Control animals were instilled with 200 μl sterile saline containing 0.2 % Tween 80.

### Acute asthma model

In the acute protocol, all animals were sensitized to ovalbumin as described above, and received a single saline or ovalbumin challenge 5 weeks after sensitisation (Fig. 1a). Animals were treated with different dosages of tiotropium (0.01, 0.03, 0.1 and 0.3 mM, 3 min inhalation time), ciclesonide (0.001, 0.01, 0.1 and 1 mg/kg) or the combination of tiotropium and ciclesonide (0.1 mM and 1 mg/kg, respectively) before ovalbumin challenge (Additional file 1: Table S1). Twenty five hours after the ovalbumin challenge, animals were anaesthetized with 20 mg/ml Brietal-sodium, 35 mg/kg ketamine hydrochloride and 6 mg/kg Sedamun intraperitoneally, which ensured a fast, deep anaesthesia. The lungs were gently lavaged with 5 ml of sterile saline at 37 °C using a tracheal cannula, followed by three subsequent aliquots of 8 ml of saline. The recovered samples were placed on ice and centrifuged at 290 *g* for 10 min at 4 °C. The combined pellets were resuspended to a final volume of 1.0 ml in PBS, and total cell numbers were counted using a coulter counter (Casy Rock). For cytological examination, cytospin preparations were stained with May-Grünwald and Giemsa stain (Sigma Chemical, St. Louis). A cell differentiation was performed by counting at least 400 cells in duplicate.

### Chronic asthma model

In the chronic protocol, all animals were sensitized to ovalbumin as described above, and 5 weeks later received saline or ovalbumin challenges once weekly for 12 weeks (Fig. 1b). Animals were treated with different dosages of tiotropium and/or ciclesonide prior to each challenge. In the first chronic dose-finding study, tiotropium nebulizer doses of 0.01 mM and 0.03 mM (3 min inhalation time) and ciclesonide doses of 0.01 mg/kg and 0.1 mg/kg were tested. See Additional file 1: Table S2 for an overview of the experimental groups included (6 groups, 4 animals per group). Based on these results, 0.01 mM tiotropium and 0.01 mg/kg ciclesonide were selected for follow-up studies investigating interactions between both drugs. See Additional file 1: Table S3 for an overview of the experimental groups included in this study (6 groups, 8 animals per group). Twenty-four hours after the last challenge, guinea pigs were sacrificed by experimental concussion, followed by rapid exsanguination. Lungs were inflated with a fixed amount (6 mL) of saline:tissue tek solution, which was gently instilled into the lungs. The lungs were immediately resected and kept on ice for further processing. Transverse frozen cross-sections of the main bronchi in the right lung lobes were used for histological and immunohistochemical analyses as described previously [22]. To optimally preserve the lungs for these histological analyses, no bronchoalveolar lavage was performed prior to the lung resection in this chronic study. To identify eosinophils, sections were stained with haematoxylin and eosin (Sigma Chemical, St. Louis). To identify smooth muscle, sections were stained for smooth muscle-myosin heavy chain (sm-MHC; Neomarkers; Fremont, CA, USA) and visualised using an HRP-linked secondary antibody, diaminobenzidine (0.3 mg/ml). Negative control staining without primary antibody was performed to demonstrate specificity. To identify collagen fibers, sections were stained with a Sirius Red stain. Airways within sections were digitally photographed and classified as cartilaginous or non-cartilaginous. The average diameter of the cartilaginous airways was 2654.6 ± 1275.8 μm and the average diameter of the non-cartilaginous airways was 548.0 μm ± 212.6 μm. All immunohistochemical measurements were carried out digitally by planimetry using quantification software (ImageJ). For this purpose, the digital photographs were blinded and analysed at a magnification of 40-400x. Of each animal, 2 to 4 lung sections were prepared per staining, in which a total of 2 to 6 airways of each classification were analysed. To quantify eosinophilia, the number of eosinophils in the different compartments was counted and expressed relative to basement membrane length. For quantification of smooth muscle mass, smooth muscle-myosin positive area in the airway wall compartment was quantified and expressed relative to square of the basement membrane length.

### Statistical analysis

Data are presented as mean ± s.e. of the mean. Statistical differences between means were calculated using one-way ANOVA, followed by Holm-Sidak post hoc test versus ovalbumin-challenged saline-treated animals. Differences were considered significant at *p* < 0.05.

## Results

### Acute asthma model - inflammation

**Fig. 2:**
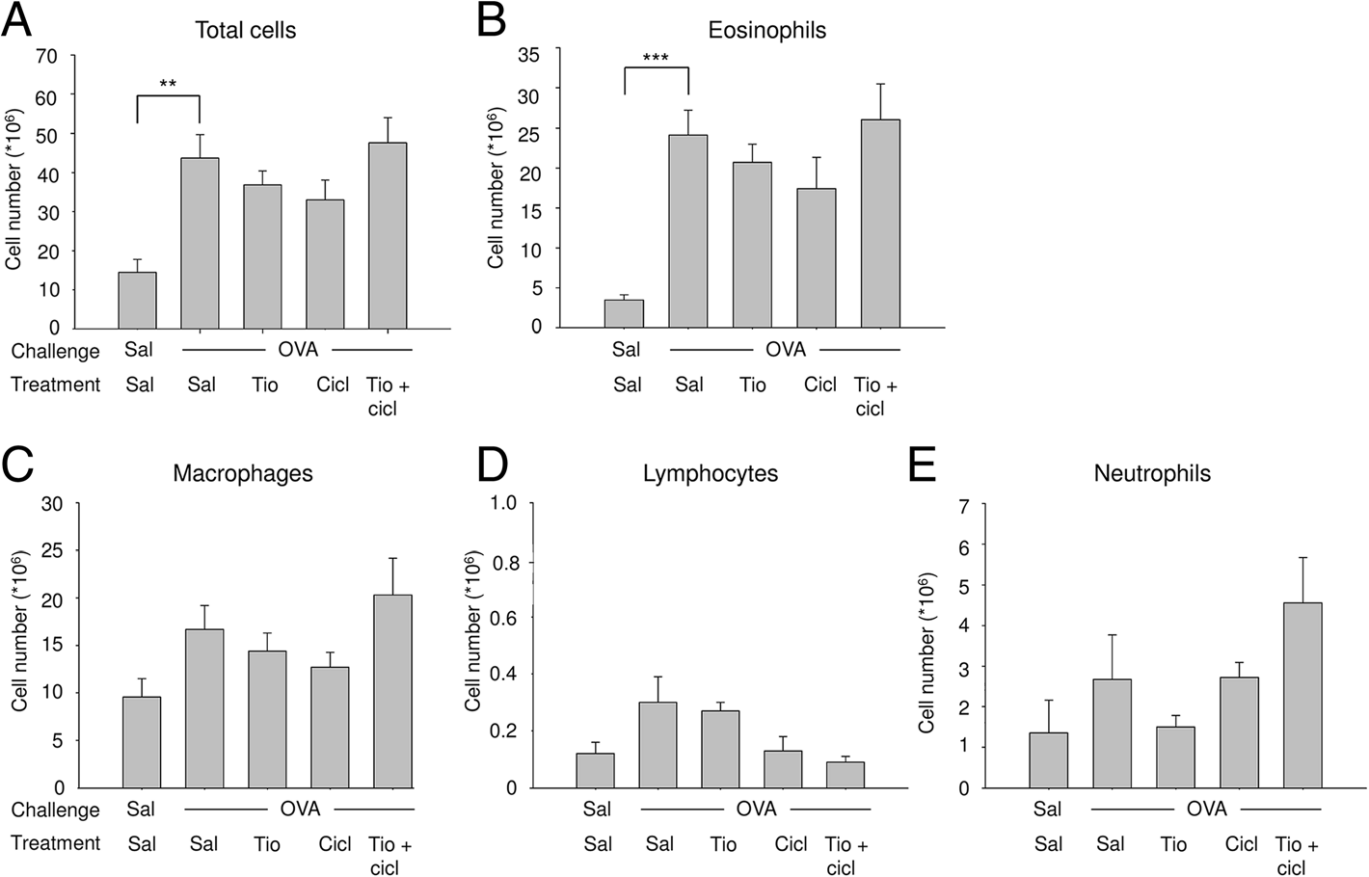
Inflammatory cell numbers in bronchoalveolar lavage fluid in response to a single ovalbumin (OVA) challenge and treatment with saline (sal; control), tiotropium (tio; 0.1 mM; nebulizer concentration), and/or ciclesonide (cicl; 1 mg/kg). Guinea pigs were treated as described in Fig. 1a. A bronchoalveolar lavage was performed 25 h after OVA challenge and inflammatory cells were determined. **a** total cells, **b** eosinophils, **c** macrophages, **d** lymphocytes, **e** neutrophils. ** *p* < 0.01, *** *p* < 0.001. Data represent mean ± s.e.m. of 5–8 animals per group

Ovalbumin challenge induced a 3-fold increase in inflammatory cell number in the broncho-alveolar lavage fluid (BALF) (Fig. 2a), consisting mostly of eosinophils (Fig. 2b). In addition, there was a small but not significant increase in macrophages (Fig. 2c), lymphocytes (Fig. 2d) and neutrophils (Fig. 2e). Treatment with tiotropium (0.1 mM; the dose used in previous studies [21, 22]) had no significant effect on allergen-induced increases in total cell number, eosinophils, macrophages, neutrophils or lymphocytes (Fig. 2). Ciclesonide treatment (1 mg/kg) also had no significant effect on inflammatory cell numbers in the BALF (Fig. 2). The combination of tiotropium and ciclesonide was not more effective than the monotherapies and no significant anti-inflammatory effects were observed in this acute asthma model, although the number of lymphocytes was repressed to the level observed in saline-challenged animals by the combination of tiotropium and ciclesonide (Fig. 2d). The same is true for the other doses of tiotropium (0.01–0.3 mM) and ciclesonide (0.001–0.1 mg/kg) assessed as part of this initial study (data not shown).

### Dose-finding in chronic asthma model - inflammation

**Fig. 3:**
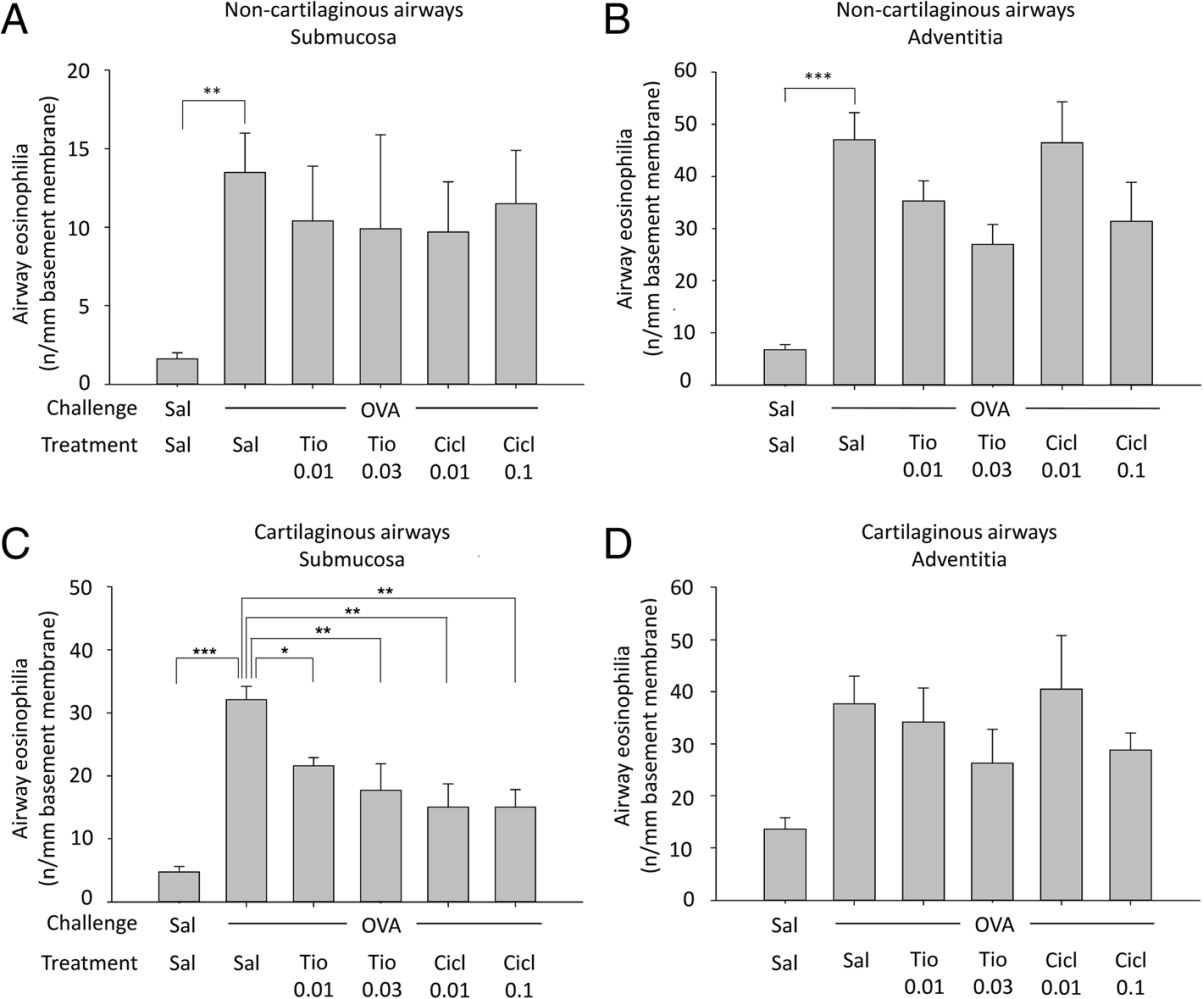
Airway eosinophilia in response to chronic ovalbumin (OVA) challenge and treatment with saline (sal; control), tiotropium (tio; 0.01 and 0.03 mM; nebulizer concencenrations) or ciclesonide (cicl; 0.01 and 0.1 mg/kg). Guinea pigs were treated as described in Fig. 1b. Lungs were collected 24 h after the last OVA challenge and eosinophil numbers were determined by H&E staining in the submucosa (**a**,**c**) and adventitia (**b**,**d**) of the non-cartilaginous (**a**,**b**) and cartilaginous airways (**c**,**d**). * *p* < 0.05, ** *p* < 0.01, *** *p* < 0.001. Data represent mean ± s.e.m. of 4 animals per group

As we have previously demonstrated profound anti-inflammatory effects of tiotropium (0.1 mM) and budesonide (0.1 mM) in a guinea pig model of chronic asthma [22], we concluded that the acute model is not predictive for the chronic situation. Therefore, an additional dose-finding study was planned in which the effects of low to moderate doses of tiotropium (0.01 and 0.03 mM) and ciclesonide (0.01 and 0.1 mg/kg) were evaluated in a model of chronic asthma in order to select doses that cause submaximal effects. In contrast to the findings in the acute model, both tiotropium and ciclesonide exerted anti-inflammatory effects in the chronic allergen model. Ovalbumin challenge induced an increase in the number of eosinophils in the submucosa (8.4-fold, Fig. 3a) and adventitia (7.0-fold, Fig. 3b) of non-cartilaginous airways, and in the submucosa (6.8-fold, Fig. 3c) and adventitia (2.8-fold, Fig. 3d) of the cartilaginous airways, although the increase in the latter was not significant. Airway eosinophilia was reduced dose-dependently by both tiotropium (15–53 % inhibition) and ciclesonide (2–62 % inhibition) treatment, and this inhibitory effect was the most profound in the submucosa of cartilaginous airways (Fig. 3c).

### Dose-finding in chronic asthma model - remodeling

**Fig. 4:**
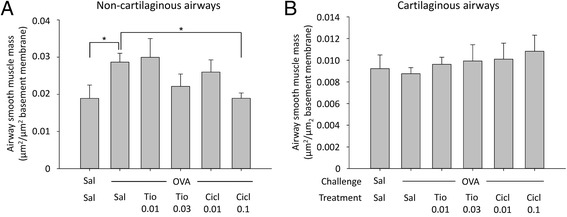
Airway smooth muscle mass thickening in response to chronic ovalbumin (OVA) challenge and treatment with saline (sal, control), tiotropium (tio; 0.01 and 0.03 mM; nebulizer concentration) or ciclesonide (cicl; 0.01 and 0.1 mg/kg). Guinea pigs were treated as described in Fig. 1b. Lungs were collected 24 h after the last OVA challenge and airway smooth muscle mass was determined by α-sm-myosin antibody staining of non-cartilaginous (**a**) and cartilaginous airways (**b**). * *p* < 0.05. Data represent mean ± s.e.m. of 4 animals per group

To evaluate the effects of tiotropium and ciclesonide on airway remodeling, airway smooth muscle mass was determined. In line with previous findings, repeated allergen challenges induced airway smooth muscle thickening in the non-cartilaginous airways (1.3-fold increase, Fig. 4a), but not the cartilaginous airways (Fig. 4b). Airway smooth muscle thickening was reduced by both tiotropium and ciclesonide treatment, although in the small number of animals used for the dose finding this was significant for the animals treated with 0.1 mg/kg ciclesonide only (100 % inhibition, Fig. 4a). In line with findings on airway eosinophilia, the highest doses of tiotropium and ciclesonide appeared to be more effective (Fig. 4a). No remodeling of the pulmonary microvasculature was observed. The number of muscularized microvessels in the cartilaginous airways was not changed in response to repeated allergen challenges, nor was there any effect of tiotropium or ciclesonide treatment.

### Drug combination in chronic asthma model – inflammation

**Fig. 5:**
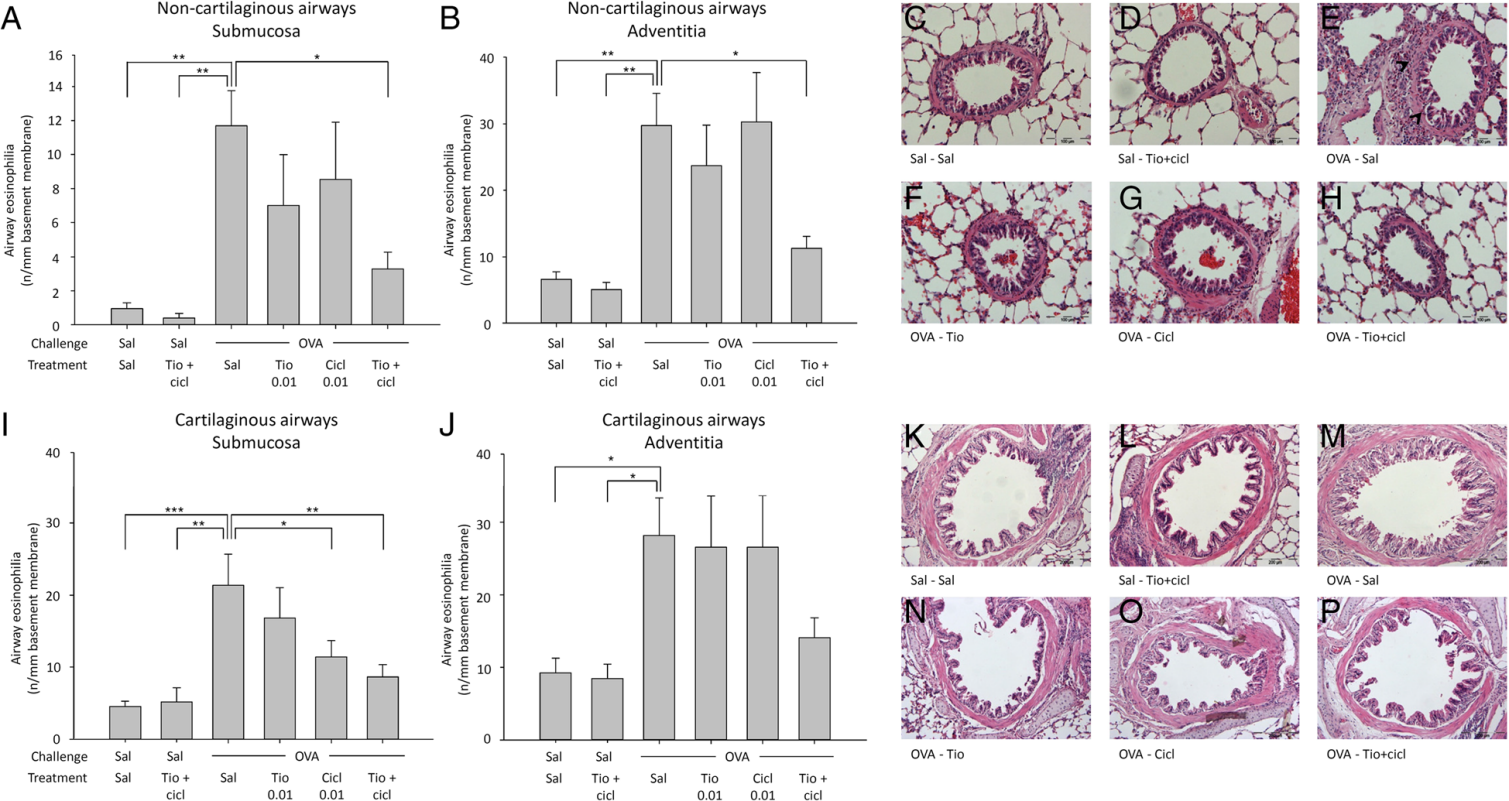
Airway eosinophilia in response to chronic ovalbumin (OVA) challenge and treatment with saline (sal; control), tiotropium (tio; 0.01 mM; nebulizer concentration) and/or ciclesonide (cicl; 0.01 mg/kg). Guinea pigs were treated as described in Fig. 1b. Lungs were collected 24 h after the last OVA challenge and eosinophil numbers were determined by H&E staining in the submucosa (**a**, **i**) and adventitia (**b**,**j**) of non-cartilaginous (**a**,**b**) and cartilaginous (**i**,**j**) airways. Representative images are shown in panels **c**-**h** for non-cartilaginous airways (magnification 200x) and in panels K-P for cartilaginous airways (maginification 100x). * *p* < 0.05, ** *p* < 0.01. Data represent mean ± s.e.m. of 8 animals per group

Based on the results of the dose-finding study in the chronic asthma model, 0.01 mM tiotropium and 0.01 mg/kg ciclesonide were selected for the follow-up study in which a combination of both drugs was investigated. These doses were selected since in most cases they produced only threshold effects on inflammation and remodeling by themselves. In line with the dose-finding study, ovalbumin challenge induced airway eosinophilia in the submucosa (12.2-fold, Fig. 5a) and adventitia (4.5-fold, Fig. 5b) of non-cartilaginous airways, and in the submucosa (4.7-fold, Fig. 5i) and adventitia (3.1-fold, Fig. 5j) of the cartilaginous airways. The combination of tiotropium and ciclesonide had no effect on eosinophil numbers in saline-challenged animals. Tiotropium (0.01 mM) did not significantly affect eosinophil numbers in the submucosa or adventitial compartments of both the non-cartilaginous airways and the cartilaginous airways (Fig. 5a, b, i and j). Similar effects were observed for ciclesonide (0.01 mg/kg), which inhibited eosinophilia in the submucosa of cartilaginous airways only (Fig. 5i). Combined treatment with tiotropium and ciclesonide had profound anti-inflammatory effects compared to ovalbumin-challenged animals, inhibiting airway eosinophilia by 74 to 80 % in all compartments (Fig. 5).

### Drug interaction in chronic asthma model – remodeling

**Fig. 6:**
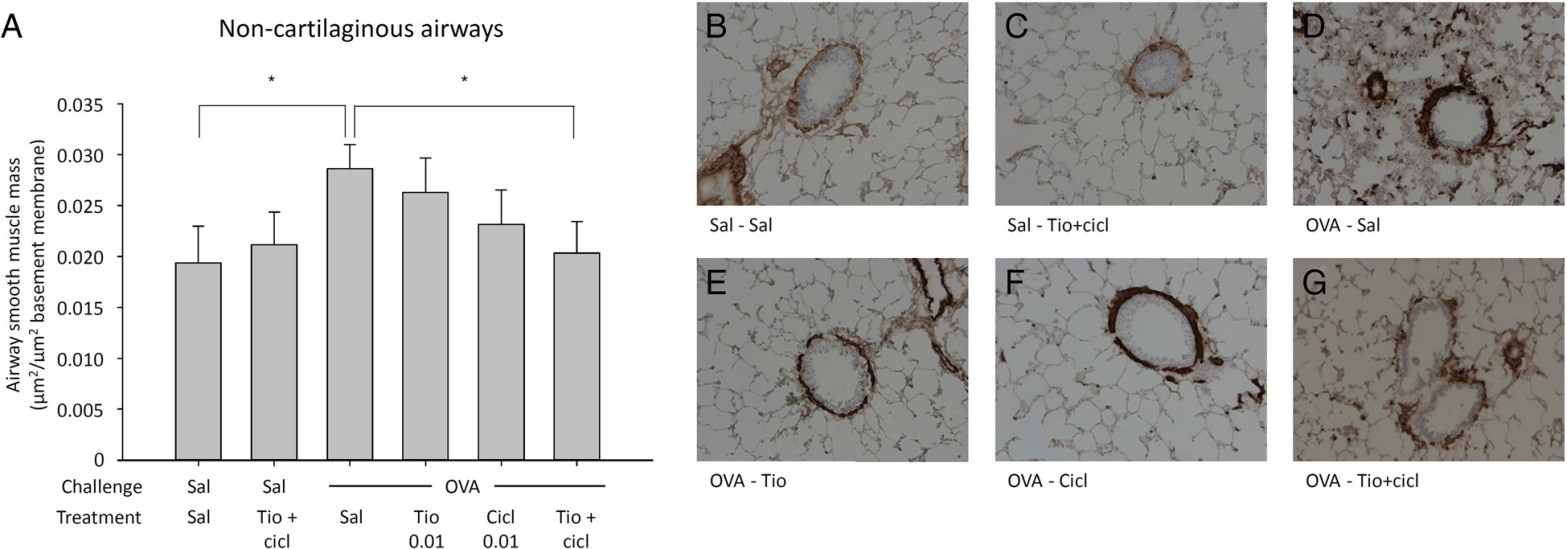
Airway smooth muscle mass thickening in response to chronic ovalbumin (OVA) challenge and treatment with saline (sal; control), tiotropium (tio; 0.01 mM; nebulizer concentration) and/or ciclesonide (cicl; 0.01 mg/kg). Guinea pigs were treated as described in Fig. 1b. Lungs were collected 24 h after the last OVA challenge and airway smooth muscle mass was determined by α-sm-myosin antibody staining. Quantification is shown in figure **a** for non-cartilaginous airways and representative images are shown in panels **b**-**g** (magnification 200x). * *p* < 0.05. Data represent mean ± s.e.m. of 8 animals per group

To assess the effect of the combination of tiotropium and ciclesonide on airway remodeling, we analysed collagen deposition and airway smooth muscle mass in response to ovalbumin challenge. As described previously for this model, no differences in airway collagen content in response to allergen exposure were observed [22], and tiotropium or ciclesonide treatment had no effect on collagen deposition either (data not shown). In line with the results from the dose-finding study, ovalbumin challenge induced an increase in airway smooth muscle mass in the non-cartilaginous airways compared to saline-challenged animals (1.5-fold, Fig. 6), but had no effect on smooth muscle mass in the cartilaginous airways (data not shown). The combination of tiotropium and ciclesonide had no effect on airway smooth muscle mass in saline-challenged animals. Tiotropium and ciclesonide alone did not significantly inhibit ovalbumin-induced airway smooth muscle mass, whereas the combination of tiotropium and ciclesonide significantly inhibited ovalbumin-induced airway smooth muscle mass by 81 % (Fig. 6).

## Discussion

The results of this study indicate that in vivo, tiotropium and ciclesonide do not protect against *acute* allergen-induced inflammation, but do protect against *chronic* allergen-induced airway inflammation and remodeling. Whereas there was only limited inhibition of airway eosinophilia and airway smooth muscle thickening after treatment with the monotherapies at threshold doses, allergen-induced alterations were significantly inhibited by pretreatment with the combination of both compounds. This suggests that combination therapy with tiotropium and ciclesonide might have beneficial effects on airway inflammation and remodeling. The potential for a steroid-sparing effect needs additional studies.

To our knowledge, this is the first study demonstrating functional interactions of anticholinergics and corticosteroids on inflammation and remodeling in vivo. Previously, it has been shown in mice and guinea pigs that monotherapy with higher doses of tiotropium or corticosteroids can inhibit allergen-induced inflammation and remodeling [21, 22, 25]. Tiotropium and dexamethasone alone, both at a dose of 1 mg/kg, inhibit airway inflammation in response to ovalbumin in mice [25]. Using the chronic guinea pig model as described in this study, we reported inhibitory effects of tiotropium and budesonide on inflammation and remodeling at 0.1 mM [21, 22]. The effects observed on airway eosinophilia in the latter study were comparable to the effects observed with the highest dose in the current study (0.03 mM tiotropium and 0.1 mg/kg ciclesonide), suggesting that the inhibitory effects occur at much lower doses than previously thought, and that approximately 50 % inhibition of airway eosinophilia by these treatments is the maximal effect that can be achieved with anticholinergic or corticosteroid treatment in this model. In this study, we demonstrate that tiotropium and ciclesonide at even lower doses of 0.01 mM and 0.01 mg/kg respectively, significantly inhibit airway inflammation and remodeling when administered in combination, with no effect on inflammation or remodeling by the monotherapies at these doses.

Synergistic effects between anticholinergics and corticosteroids have also been observed in vitro. It has been shown that glycopyrrolate alone does not affect TNF-alpha release from monocytes, but synergistically enhances the inhibitory effects of budesonide on TNF-alpha release [23]. This synergy between anticholinergics and corticosteroids on inflammatory processes is now confirmed by our in vivo findings. Randomized clinical trials investigating the effects of long-acting anticholinergics in asthma have only recently attracted attention, and there is no study that has investigated synergism between anticholinergics and corticosteroids in patients with asthma. However, beneficial effects of combining anticholinergics and corticosteroids have been reported. The addition of tiotropium to treatment for patients with uncontrolled asthma was shown to be more effective on improving asthma symptoms and lung function than doubling the dose of corticosteroids [26]. Improvements in morning peak expiratory flow, the proportion of asthma-control days, forced expiratory volume in 1 s and daily symptom scores were reported in this crossover trial [26]. Furthermore, randomized controlled clinical trials demonstrated that treatment with tiotropium, added on to ICS or ICS plus LABA, is an effective therapy for moderate and severe asthma patients as seen by improvements in lung function and reduction in the risk of severe asthma exacerbations [5–7], indicating at least additive effects of tiotropium when added to ICS +/− LABA treatment.

The mechanistic basis for functional interactions between anticholinergics and corticosteroids is not yet clear, but it may well be that these drugs target specific and distinct pathophysiological processes. Ciclesonide, via glucocorticosteroid receptors, acts anti-inflammatory by repressing pro-inflammatory gene transcription [27]. These mechanisms are most likely different from those targeted by tiotropium, which acts via G-protein coupled muscarinic receptors. In vitro evidence exists for anti-inflammatory and anti-remodeling effects of anticholinergics on airway cells via muscarinic receptors [8]. Anticholinergics inhibit the release of neutrophil chemotactic mediators from a number of cells, including macrophages, fibroblasts, airway smooth muscle cells and epithelial cells [11-13]. Anticholinergics also inhibit parameters of remodeling, including enhanced MUC5AC expression, goblet cell metaplasia, and fibroblast to myofibroblast transition [16, 28, 29]. In addition, bronchoconstriction is effectively targeted by tiotropium, and this may also have its effects on airway inflammation and remodeling [30, 31]. This hypothesis is supported by recent data showing that repeated methacholine challenges induce remodeling in mild asthma patients [32]. Moreover, we demonstrated that muscarinic M_3_ receptor knock-out mice are protected from allergen-induced airway remodeling, even though there is still an inflammatory response in these animals [20]. Because M_3_ receptors mediate bronchoconstriction, which is abolished in M_3_ receptor knock-out mice [33], this may suggest that bronchoconstriction by itself might be an important driver of airway remodeling [20, 31]. In support, we previously demonstrated that methacholine treatment promotes remodeling in guinea pig lung slices [34]. Taken together, we propose that additive effects between anticholinergics and corticosteroids, as observed for tiotropium and ciclesonide in our study, are based on the different mechanisms they target.

Surprisingly, no anti-inflammatory effects of tiotropium and ciclesonide were observed in the acute asthma model in this study. Similar findings were observed for tiotropium and the long-acting β-agonist olodaterol in an acute guinea pig model [35]. Apparently, chronic treatment is needed to unmask the anti-inflammatory effects in guinea pigs, as we do observe inhibition of inflammation in this study and previous studies after multiple allergen challenges [22]. Because mast cell infiltration occurs already after the systemic sensitization against ovalbumin (i.e. prior to the first drug treatment), it may be that tiotropium cannot protect against the initial inflammatory response induced by the first allergen encounter, but requires prolonged treatment. In support, anticholinergics do not inhibit mast cell degranulation [36]. Similarly, the effects of corticosteroids require prolonged treatment, as the same discrepancy between acute and chronic inflammation was seen for ciclesonide, which was substantially more effective, and at lower doses already, in inhibiting chronic allergen-induced inflammation compared with acute allergen-induced inflammation. Limitations in the delivery of the drugs do not explain the difference, as a single administration of tiotropium effectively prevents bronchoconstriction and the early and late asthmatic reaction [35], and a single administration of corticosteroids inhibits the late asthmatic reaction in this model (unpublished observations). The mechanistic basis for the delay in the onset of action of tiotropium is unclear, however, it can be envisaged that the role of acetylcholine is further downstream in the pathophysiological process. This is supported by the fact that sensory nerves play an important role in the late asthmatic reaction, and not in the early asthmatic reaction [37]. It may therefore be that an initial inflammatory response is required, which leads to epithelial damage, inflammatory mediator release and other mechanisms that enhance the cholinergic reflex, and thereby increase the role of acetylcholine later on.

The finding that tiotropium and ciclesonide protect against allergen-induced airway smooth muscle thickening is in agreement with our previous findings [21]. The reduction in smooth muscle mass may be related to the reduction in airway inflammation, as many inflammatory mediators are reported to promote smooth muscle growth in allergic airway inflammation [38]. Alternatively, increased cholinergic activity that results from airway inflammation promotes bronchoconstriction, which might drive airway remodeling as discussed above.

## Conclusion

In conclusion, chronic treatment with a combination of low dose tiotropium and ciclesonide inhibits airway inflammation and remodeling in a guinea pig model of chronic asthma, suggesting that treatment with tiotropium and ciclesonide may have anti-inflammatory and anti-remodeling activity in allergic airway diseases. Given the fact that tiotropium is now registered as a therapy for asthma added on to ICS and LABA treatment, beneficial effects of combination therapy on these inflammatory and remodeling parameters may be clinically relevant.

## Additional file

Additional file 1: Table S1. Experimental groups included in the acute protocol. The data from group 1–5 is depicted in Fig. 2, data from the other groups is not shown. **Table S2.** Experimental groups included in the chronic dose-finding protocol (*n* = 4 animals per group). **Table S3.** Experimental groups included in the chronic protocol investigating interactions between tiotropium and ciclesonide (*n* = 8 animals per group). (DOCX 18 kb)

## Abbreviations

BALF: Bronchoalveolar lavage fluid
Cicl: Ciclesonide
ICS: Inhaled corticosteroid
LABA: Long-acting β-agonist
LAMA: Long-acting muscarinic antagonist
OVA: Ovalbumin
Sal: Saline
Tio: Tiotropium
