# Supplementary material for: Combination therapy of tiotropium and ciclesonide attenuates airway inflammation and remodeling in a guinea pig model of chronic asthma
Source: Respir Res. 2016 Feb 4;17:13. doi: 10.1186/s12931-016-0327-6 (PMC4743207; doi:10.1186/s12931-016-0327-6)
Supplement: Additional file 1: Table S1. — Experimental groups included in the acute protocol. The data from group 1–5 is depicted in Fig. 2, data from the other groups is not shown. Table S2. Experimental groups included in the chronic dose-finding protocol (n = 4 animals per group). Table S3. Experimental groups included in the chronic protocol investigating interactions between tiotropium and ciclesonide (n = 8 animals per group). (DOCX 18 kb) [file 12931_2016_327_MOESM1_ESM.docx]

**Additional file 1**

Title: Combination therapy of tiotropium and ciclesonide attenuates airway inflammation and remodeling in a guinea pig model of chronic asthma

Loes E.M. Kistemaker^1,2*^, I. Sophie T. Bos^1,2^, Mark H. Menzen^1,2^, Harm Maarsingh^3^, Herman Meurs^1,2^, Reinoud Gosens^1,2^

**^1^ Department of Molecular Pharmacology, University of Groningen, Groningen, The Netherlands.**

**^2^ GRIAC Research Institute, University Medical Center Groningen, University of Groningen, Groningen, The Netherlands Department of Pathology and Medical Biology, University Medical Centre Groningen, Groningen, The Netherlands**

**^3^ Department of Pharmaceutical Sciences, Gregory School of Pharmacy, Palm Beach Atlantic University, Florida, USA**

* Author for correspondence

Loes E.M. Kistemaker

Department of Molecular Pharmacology

University of Groningen

A. Deusinglaan 1

9713 AV Groningen

The Netherlands

Tel. + 31 50 363 6372

Fax. +31 50 363 6908

Email: l.e.m.kistemaker@rug.nl

Table S1: Experimental groups included in the acute protocol. The data from group 1-5 is depicted in figure 2, data from the other groups is not shown.

| Group | Number | Treatment (-24h) | Treatment (-1h) | Challenge |
| --- | --- | --- | --- | --- |
| 1 | 6 | Saline | Saline | Saline |
| 2 | 8 | Saline | Saline | Ovalbumin |
| 3 | 5 | Saline | Tiotropium 0.1 mM | Ovalbumin |
| 4 | 7 | Ciclesonide 1 mg/kg | Ciclesonide 1 mg/kg | Ovalbumin |
| 5 | 7 | Ciclesonide 1 mg/kg | Ciclesonide 1 mg/kg +  Tiotropium 0.1 mM | Ovalbumin |
| 6 | 4 | Saline | Tiotropium 0.01 mM | Ovalbumin |
| 7 | 4 | Saline | Tiotropium 0.03 mM | Ovalbumin |
| 8 | 4 | Saline | Tiotropium 0.3 mM | Ovalbumin |
| 9 | 3 | Ciclesonide 0.001 mg/kg | Ciclesonide 0.001 mg/kg | Ovalbumin |
| 10 | 3 | Ciclesonide 0.01 mg/kg | Ciclesonide 0.01 mg/kg | Ovalbumin |
| 11 | 4 | Ciclesonide 0.1 mg/kg | Ciclesonide 0.1 mg/kg | Ovalbumin |

Table S2: Experimental groups included in the chronic dose-finding protocol (n=4 animals per group).

| Group | Treatment (-24h) | Treatment (-1h) | Challenge |
| --- | --- | --- | --- |
| 1 | Saline | Saline | Saline |
| 2 | Saline | Saline | Ovalbumin |
| 3 | Saline | Tiotropium 0.01 mM | Ovalbumin |
| 4 | Saline | Tiotropium 0.03 mM | Ovalbumin |
| 5 | Ciclesonide 0.01 mg/kg | Ciclesonide 0.01 mg/kg | Ovalbumin |
| 6 | Ciclesonide 0.1 mg/kg | Ciclesonide 0.1 mg/kg | Ovalbumin |

Table S3: Experimental groups included in the chronic protocol investigating interactions between tiotropium and ciclesonide (n=8 animals per group).

| Group | Treatment (-24h) | Treatment (-1h) | Challenge |
| --- | --- | --- | --- |
| 1 | 12 x Saline | 12 x Saline | 12 x Saline |
| 2 | 12 x Ciclesonide 0.01 mg/kg | 12 x Ciclesonide 0.01 mg/kg  + Tiotropium 0.01 mM | 12 x Saline |
| 3 | 12 x Saline | 12 x Saline | 12 x Ovalbumin |
| 4 | 12 x Saline | 12 x Tiotropium 0.01 mM | 12 x Ovalbumin |
| 5 | 12 x Ciclesonide 0.01 mg/kg | 12 x Ciclesonide 0.01 mg/kg | 12 x Ovalbumin |
| 6 | 12 x Ciclesonide 0.01 mg/kg | 12 x Ciclesonide 0.01 mg/kg  + Tiotropium 0.01 mM | 12 x Ovalbumin |
